# ﻿Multilocus phylogeny and morphology reveal two new species of *Lepiota* (Agaricales, Verrucosporaceae) from southwestern China

**DOI:** 10.3897/mycokeys.123.163999

**Published:** 2025-10-14

**Authors:** Xing Li, Bin Chen, Yanliu Chen, Mengya An, Junfeng Liang

**Affiliations:** 1 Research Institute of Tropical Forestry, Chinese Academy of Forestry, Guangzhou 510520, China Chinese Academy of Forestry Guangzhou China; 2 Henan Institute of Science and Technology, College of Life Science, Xinxiang 453003, China College of Life Science Xinxiang China

**Keywords:** *
Lepiota
*, lepiotaceous fungi, phylogeny, taxonomy

## Abstract

Based on phylogenetic and morphological evidence, two new Lepiota sect. Lepiota species collected from southwestern China are described and illustrated, namely *L. brunneophora* and *L. ochraceosquamea*. *Lepiota brunneophora* has a pileus with yellowish-brown to brown squamules and striations, a reddish-brown to purplish-brown stipe covered with whitish, floccose squamules, and penguin-shaped basidiospores with a narrowed apex. *Lepiota ochraceosquamea* is characterized by dark yellowish-brown to light ochre squamules on the pileus, a whitish annulus, broadly fusiform basidiospores, and a trichodermal pileus covering composed of subcylindrical terminal elements, narrowing toward the apex and lacking basal short elements. A comprehensive phylogenetic framework within L. sect. Lepiota, including agaricoid and sequestrate species, is also provided.

## ﻿Introduction

*Lepiota* (Pers.) Gray is a saprotrophic genus, mainly distributed in (sub)tropical to temperate regions (Vellinga 2004a; Sysouphanthong et al. 2011). The genus was long classified in the Agaricaceae Chevall. (Lange 1935; Pegler 1986; Singer 1986; Candusso and Lanzoni 1990; Vellinga 2001; Liang 2007; Kirk et al. 2008; Yang et al. 2019) until it was recently transferred to the family VerrucosporaceaeJülich (1982) (= Lepiotaceae Kun L. Yang, Jia Y. Lin & Zhu L. Yang, see Yang et al. 2024) (Kalichman et al. 2020; Salichanh et al. 2024; Qu et al. 2025). Some fungi of this genus are non-toxic but of low edible value, such as *L. erminea* (Fr.) P. Kumm. and *L. magnispora* Murrill (Dai et al. 2010), while others are highly poisonous, such as *L. brunneoincarnata* Chodat & C. Martin and *L. subincarnata* J.E. Lange (Sgambelluri et al. 2014; Sarawi et al. 2022; Bau et al. 2024), whose amatoxins possess significant research and application value in life sciences and medical biology (Wieland and Faulstich 1991). Furthermore, some *Lepiota* species play an important role in forest ecosystems, particularly in the decomposition of organic matter (Guzmán and Guzmán-Davalos 1992; Vellinga 2004b).

However, the genus *Lepiota* is also difficult to distinguish because of its complex taxonomic characters. Vellinga (2001) carried out a comprehensive arrangement of *Lepiota* based mainly on the structure of the pileus covering and the shape of basidiospores. Her treatment was accepted by most taxonomists (Liang 2007; Yang et al. 2019; Bashir et al. 2020; Niazi et al. 2021; Kaygusuz 2022; Haqnawaz et al. 2022; Rehman et al. 2024), but not supported by her own molecular evidence, which indicated that *Lepiota* is not monophyletic (Vellinga 2003). Combining morphological and molecular phylogenetic analysis, Hou and Ge (2020) treated the species of the large-spored group within L. sect. Echinatae/*Echinoderma* s.l. as *Echinoderma*Bon (1991) s.s. and those of the small-spored group as the basal members of *Lepiota*, resulting in the monophyly of *Lepiota*. Sarawi et al. (2025) contributed to the sectional nomenclature, taxonomy, and phylogeny of *Lepiota*, dividing the genus into seven sections: L. sect. Stenosporae J.E. Lange ex Reschke & Sarawi (Sarawi et al. 2025), L. sect. Helveolae (Bon & Boiffard) Bon (1993), L. sect. Cristatae Kühner ex Wasser (1978), L. sect. Fuscovinaceae Bon & Candusso (Candusso and Lanzoni 1990), L. sect. Lepiota, L. sect. LilaceaeBon (1981), and L. sect. Eriophorae (Bon) Reschke & Sarawi (Sarawi et al. 2025).

During recent investigations, two *Lepiota* species were collected from southwestern China for further morphological comparison and phylogenetic analysis. The results led to the discovery of two new species of L. sect. Lepiota, which are described and illustrated here.

## ﻿Materials and methods

### ﻿Morphology

Specimens in this study, collected from three provinces or autonomous regions, viz., Yunnan, Xizang, and Sichuan, were deposited in the Herbarium of the Research Institute of Tropical Forestry, Chinese Academy of Forestry (RITF), and the Herbarium of Cryptogams, Kunming Institute of Botany, Chinese Academy of Sciences (HKAS). Macromorphological features were directly recorded in the field, with color photos of basidiomata and measurements of the pileus, stipe, etc., taken with a ruler (0.5 mm scale). Fresh basidiocarps were stored in sealed bags with silica gel for DNA extraction. The descriptive terms and herbarium acronyms follow Vellinga (1988) and Thiers (2025), respectively. Color codes follow Kornerup and Wanscher (1981).

The material preparation, methods of chemical reactions, and measurement and statistics of micromorphological characters all follow Yang et al. (2019). Sections (20–50 μm) of specimens obtained by manual slicing were fixed in 5% KOH, Congo red, or Melzer’s reagent for microscopic observation. Basidiospores were placed in cresyl blue to test their metachromatic reaction. At least 20 elements were randomly measured from each character per collection. All features and dimensions of basidia, basidiospores, cheilocystidia, and the elements of the pileus covering were observed and measured with a ZEISS Imager M2 microscope at 1000× magnification. The abbreviation [n/m/p] indicates the measurement of n basidiospores from m basidiocarps in p specimens. The length or width of basidiospores is expressed by the notation (a)b–c(d), where 90% of the measured values fall between b and c, and a and d represent the minimum and maximum values, respectively. The length-to-width ratio of the basidiospore is denoted by Q, and **Q** (in bold) represents the average Q and standard deviation of all measured basidiospores.

### ﻿DNA extraction, amplification, and sequencing

Using the modified CTAB approach (Li et al. 2013), total genomic DNA was extracted from silica-dried samples. Four DNA loci were amplified with different primers in the polymerase chain reaction (PCR), as follows: ITS1F (Gardes and Bruns 1993) and ITS4 (White et al. 1990) for the internal transcribed spacer region (ITS); LR0R and LR7 for the large subunit (LSU) of the ribosomal DNA (Vilgalys and Hester 1990); LR12R and 5SRNA for the intergenic spacer (IGS) regions (White et al. 1990); and MS3 and MS4 for the mitochondrial small ribosomal RNA subunit (mtSSU) regions (Liang et al. 2009). PCR products were delivered to Novo Gene Company (Beijing, China) for purification, sequencing, and editing.

### ﻿Phylogenetic analysis

In the phylogeny, a total of 66 representatives (including all sequenced and described species of L. sect. Lepiota reported by previous studies) were sampled, with *L. subcastanea* Jun F. Liang & Zhu L. Yang and *L. mandarina* Jun F. Liang & Zhu L. Yang (Liang 2016) from L. sect. Stenosporae as outgroup. The available sequences were uploaded to GenBank and are listed in Suppl. material 1 together with all sampled taxa and voucher information. Using MAFFT v7.490 (Katoh and Standley 2013), four DNA loci were aligned and concatenated into a single data matrix (ITS–LSU–IGS–mtSSU). Phylogenetic reconstructions were performed using Maximum Likelihood (ML) and Bayesian Inference (BI) tools implemented in the PhyloSuite v1.2.3 platform (Xiang et al. 2023; Zhang et al. 2020). ModelFinder v2.2.0 (Kalyaanamoorthy et al. 2017) was used to select the best-fit partition model (edge-linked) for ML and BI analyses under the Bayesian Information Criterion (BIC). The best-fit partition models were as follows: IGS (ML: K3Pu+F+I+G, BI: HKY+F+I+G4), ITS (ML: TPM2u+F+G4, BI: HKY+F+G4), LSU (ML: K2P+R2, BI: K2P+I+G4), and mtSSU (ML: TVM+F+R2, BI: GTR+F+I+G4). Maximum likelihood phylogenies were inferred with IQ-TREE v2.2.0 (Nguyen et al. 2015), with 5000 ultrafast bootstrap replicates (Minh et al. 2013) and 1000 SH-aLRT tests (Guindon et al. 2010) to assess branch supports. Bayesian inference phylogenies were conducted in MrBayes v3.2.7 (Ronquist et al. 2012), employing two parallel runs of 40 million generations each (sample frequency once every 1000 generations), with the first 25% discarded as burn-in. ML and BI trees were visualized in FigTree v1.4.4 (http://tree.bio.ed.ac.uk/software/figtree/).

## ﻿Results

### ﻿Phylogenetic analysis

**Figure 1.**
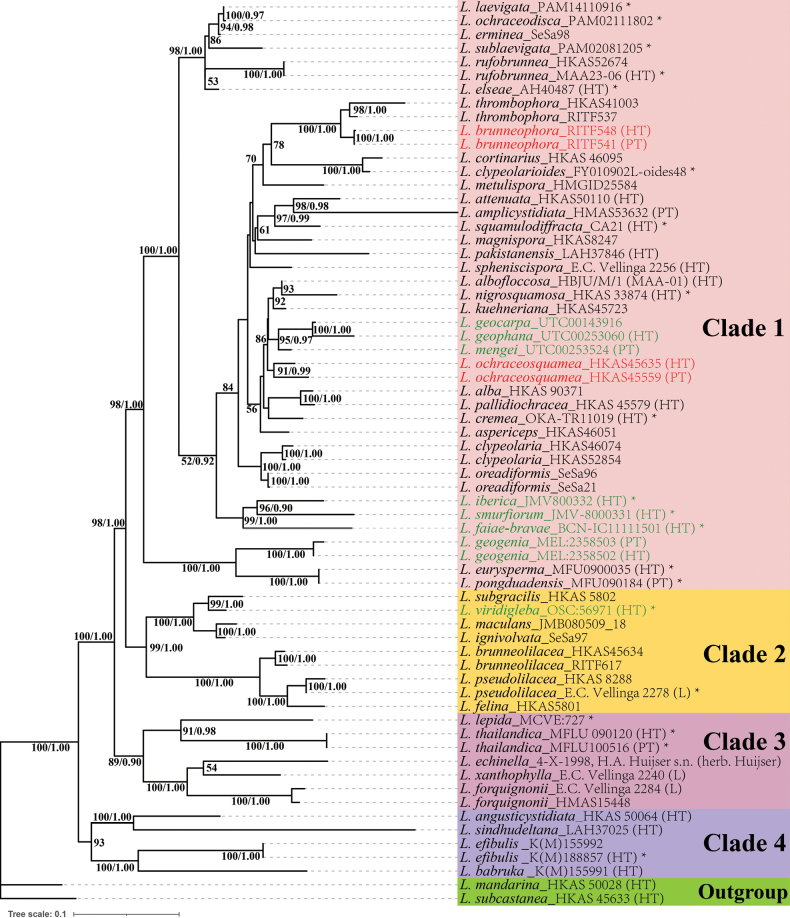
Phylogenetic tree derived from both ML and BI methods based on combined ITS-LSU-IGS-mtSSU data, showing the phylogenetic position of two new L. sect. Lepiota species (red font) and sequestrate species (green font). Bootstrap values (≥50%) and posterior probabilities (≥0.90) are indicated at each node. An asterisk indicates the sample for which only the ITS sequence is available.

The combined data matrix (ITS–LSU–IGS–mtSSU) had an alignment (Suppl. material 2) of 4,726 bp (IGS = 1–1210, ITS = 1211–2381, LSU = 2382–3838, mtSSU = 3839–4726), including sequence divergence of 1,641 variable sites (34.72%), comprising 641 singleton variable sites (13.56%) and 1,000 parsimony-informative sites (21.16%). The topologies of both ML and BI trees were similar, and the ML tree was chosen for visualization (Fig. 1), with nodal support values from both methods indicated at the corresponding nodes. As shown in the phylogenetic tree, four main clades could be recognized. Two new species each formed a well-supported, monophyletic clade within Clade 1 and were distantly related to known and sequenced species, viz., *L. brunneophora* (BS = 100%, PP = 1.00) and *L. ochraceosquamea* (BS = 91%, PP = 0.99). *L. brunneophora* was sister to *L. thrombophora* (Berk. and Broome) Sacc., together forming a monophyletic lineage with strong support (BS = 100%, PP = 1.00). *L. ochraceosquamea* is grouped with some species (BS = 86%), including sequestrate species (*L. geocarpa* Vellinga & T. Lebel, *L. geophana* Vellinga & T. Lebel, *L. mengei* (Kropp & Castellano) T. Lebel & Vellinga) and agaricoid species (*L. albofloccosa* M. Ahamed, A.K. Dutta, K. Verma & Y.P. Sharma, *L. kuehneriana* Locq., *L. nigrosquamosa* Jun F. Liang & Zhu L. Yang). Additionally, sequestrate species with available sequences and formal descriptions all phylogenetically fell into L. sect. Lepiota. These species include *L. iberica* J.M. Vidal & Juste, *L. smurfiorum* J.M. Vidal & F. García, *L. geogenia* T. Lebel & Vellinga, *L. viridigleba* (Castellano) Z.W. Ge, Castellano & M.E. Sm., *L. faiae-bravae* Chautrand, A. Paz & Lavoise, and the three sequestrate species mentioned above, most of which have globose to subglobose basidiospores. In addition, *L. pallidiochracea* Jun F. Liang & Zhu L. Yang was nested within Clade 1 and sister to *L. alba* (Bres.) Sacc. (BS = 100%, PP = 1.00). Interestingly, *L. eurysperma* Sysouph., K.D. Hyde & Vellinga and *L. pongduadensis* Sysouph., K.D. Hyde & Vellinga clustered into a small, strongly supported clade (BS = 100%, PP = 1.00) with equal branch length.

### ﻿Taxonomic treatment

#### ﻿ Lepiota brunneophora

Taxon classificationFungiAgaricalesVerrucosporaceae

J. F. Liang & X. Li, sp. nov .

A17A7C8F-1259-52B7-B477-D0825F6F206A

859649

Fig. 2

##### Holotype.

China • Yunnan: Lijiang City, Jinshan Town, Tuanshan Reservoir, ca. 2500 m a.s.l., 18 July 2008, *Liang 931* (RITF548).

##### Etymology.

‘brunneophora’ refers to the brown color of the stipe.

##### Diagnosis.

*Lepiota brunneophora* is characterized by reddish-brown to purplish-brown stipe covering whitish floccose squamules, penguin-shaped basidiospores with papilliform apex when shrinking, broadly clavate cheilocystidia, and a pileus covering with a layer of short elements.

##### Description.

**Figure 2.**
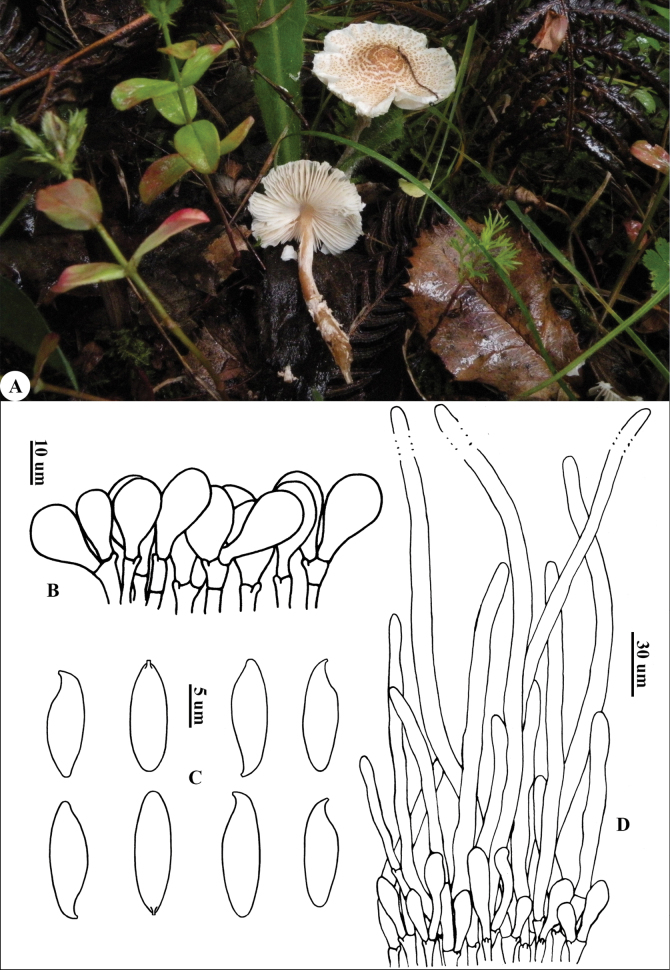
*Lepiota brunneophora* (holotype, RITF548) A. Basidiomata; B. Cheilocystidia; C. Basidiospores; D. Pileus covering. Both the photo and the line drawing are by Junfeng Liang.

***Basidiomata*** small to medium (Fig. 2A). ***Pileus*** 2.3–6.0 cm in diam, plano-convex or applanate with blunt umbo in the center, surface dry, white to cream, with yellowish-brown (6D5–6D6) to brown squamules, tearing from the center to the periphery into rings, accompanied by growth, margin upturned and striate, fragile and easily breaking off. ***Context*** whitish, thin. ***Lamellae*** L = 40–60, l = 1–2, free, whitish, moderately crowded, length unequal. ***Stipe*** 2.5–8.0 × 0.4–0.6 cm, cylindrical, hollow, thickening towards the base, reddish-brown to purplish-brown (6D6–6D7), covered with whitish and floccose squamules, yellowish-brown (6D5–6D6) squamules at the base. ***Annulus*** whitish, membranous, evanescent. Odor not distinct. Taste not recorded. Spore print white.

***Basidiospores*** (Fig. 2C) [40/2/2] 12.5–14.0(14.5) × 4.0–4.5(5.0) µm [Q = (2.50)2.77–3.5(3.63), Q = 3.08 ± 0.28], penguin-shaped in side view, with suprahilar depression, adaxial side convex, abaxial side nearly straight, apex contracted and narrowed, papilliform, fusiform in front view; colorless, hyaline, smooth, dextrinoid, slightly thick-walled, congophilous, not metachromatic in cresyl blue. ***Basidia*** 20–27 × 8–12 µm, clavate, 4-spored. Lamella edge sterile. ***Cheilocystidia*** (Fig. 2B) 9–23 × 10–24 µm, mostly clavate to sub-spherical, apex obtuse; colorless, hyaline, thin-walled. ***Pleurocystidia*** absent. ***Pileus covering*** (Fig. 2D) a trichoderm consisting of elongate, subcylindrical, apically attenuate, terminal elements 120–450 × 8–10 µm, without or rarely with septa at the base, base mixed with a layer of short and clavate elements (35–90 × 7–11 µm), with yellow-brownish intracellular pigment. ***Clamp connections*** present in all tissues.

##### Distribution.

Known only from Yunnan Province, China.

##### Habitat.

Solitary or in small groups, saprotrophic and terrestrial on grasslands under the mixed conifer and broadleaf forest in summer.

##### Additional specimens examined.

China • Yunnan: Jinghong City, Dadugang Town, 7 September 2007, alt. 1050 m, *Liang 808* (RITF541, paratype).

##### Notes.

*Lepiota brunneophora* is characterized by its pileus with yellowish-brown to brown squamules and striate margin, reddish-brown to purplish-brown stipe covering with whitish and floccose squamules, penguin-shaped basidiospores with a distinctly narrowed apex, clavate to sub-spherical cheilocystidia, and a trichodermal pileus covering intermixed with clavate and short elements.

*Lepiota brunneophora* is similar to *L. attenuata* Jun F. Liang & Zhu L. Yang in its small basidiomata, the absence of an annulus, a pileus with yellowish-brown squamules and a striate margin, and penguin-shaped basidiospores with a distinctly narrowed apex, but the difference between the two lies in that the latter has whitish stipe covering with fine grayish orange squamules, longer basidiospores, and a pileus covering with inflated elements (Liang et al. 2011).

*Lepiota metulispora* (Berk. & Broome) Sacc. and *L. pakistanensis* A. Rehman, Afshan, Usman & Khalid have a pileus with pale brown to brown squamules and striations. Both species have some features that serve as diagnostic characteristics separating them from *L. brunneophora*, such as *L. metulispora* having a whitish annulus, stipe covering with tomentose squamules at the lower part, penguin-shaped basidiospores without a distinctly narrowed apex (Liang et al. 2011), while *L. pakistanensis* possesses a whitish annulus, stipe covering with creamy white squamules at the basal part, and penguin-shaped basidiospores without a distinctly narrowed apex (Rehman et al. 2024).

Besides, *L. cortinarius* J.E. Lange could be distinguished from *L. brunneophora* by larger basidiomata, a pileus without striation on the margin, whitish stipe with pale yellow to dark brown squamules, and basidiospores without a narrowed apex (Lange 1915; Liang 2007).

*Lepiota clypeolarioides* Rea was typically different from *L. brunneophora* in its whitish to pale ochre pileus without striation, a stipe with whitish upper part and dirty white part, and smaller (6–8 × 4–5 μm) and ellipsoid basidiospores (Rea 1922; Hausknecht and Pidlich-Aigener 2005).

*Lepiota ampliocystidiata* Jun F. Liang shares some identical features with *L. brunneophora*, such as small basidiomata, pileus with striation, penguin-shaped basidiospores with a distinctly narrowed apex, but is differentiated by its dark brown to black brownish squamules on the pileus, whitish annulus, longer [(13.0) 15.0–20.0 (21.5) × (4.0) 4.5–5.5 (6.0) μm] basidiospores (Liang 2012).

Phylogenetic analysis indicated that *L. brunneophora* is sister (BS = 100% & PP = 1.00) to *L. thrombophora*. Unlike the former, the latter exhibits reddish-brown to dark brown squamules on the pileus, stipe displaying cream and smooth above the annulus and pale brown below, whitish annulus, penguin-shaped basidiospores without a distinctly narrowed apex (Saccardo 1887; Zhou 2010; Liang et al. 2011).

#### ﻿ Lepiota ochraceosquamea

Taxon classificationFungiAgaricalesVerrucosporaceae

J. F. Liang & Zhu L. Yang, sp. nov .

C4CD7860-43A7-56EC-97BD-0572A1EA9ECE

859650

Fig. 3

##### Holotype.

China • Xizang: near Yikang County, 23 July 2004, alt. 3500 m, *Yang 4173* (HKAS45559).

##### Etymology.

‘>ochraceosquamea’ refers to the color of squamules on the pileus surface.

##### Diagnosis.

*Lepiota ochraceosquamea* is characterized by whitish pileus covering with dark yellowish-brown to light ochre squamules, smooth stipe possessing fine and brown squamules at the base, broadly fusiform or oblong basidiospores (8.5–11.0 × 5.0–6.5 μm), diverse cheilocystidia (clavate, fusiform, or falcate), pileus covering a trichoderm consisting of long, erect elements, without basal short elements.

##### Description.

**Figure 3.**
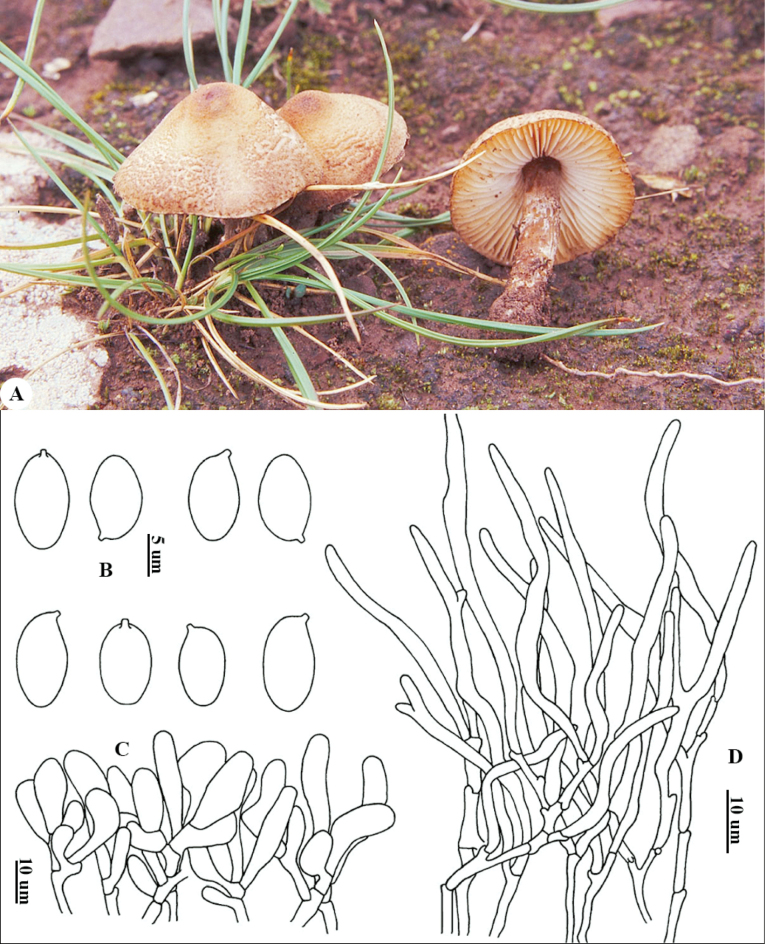
*Lepiota ochraceosquamea* (holotype, HKAS45559) A. Basidiomata; B. Basidiospores; C. Cheilocystidia; D. Pileus covering. Photo and line drawing by Zhu L. Yang and Junfeng Liang, respectively.

***Basidiomata*** small. ***Pileus*** 1–3 cm in diam, initially campanulate, extended gradually, accompanied by development, plano-convex, surface covering with dark yellowish-brown to light ochre (6D7–6D8) squamules on a whitish surface, with blunt and brown (6D8) umbonate center. Context whitish, thin. ***Lamellae*** L = 40–60, l = 1–2, free, whitish, then turning dirty white, moderately crowded, ventricose, length unequal. ***Stipe*** 1.5–3.5 × 0.2–0.4 cm, tapering upwards, nearly brown, glabrous, with fine and brown (6D6–6D7) squamules at the base. ***Annulus*** whitish, margin nearly brown, and evanescent (Fig. 3A). Smell not distinct; taste not recorded.

***Basidiospores*** (Fig. 3B) [67/3/3] (8.0)8.5–11.0(11.5) × 5.0–6.5 μm [Q = (1.45) 1.50–2.00, Q = 1.68 ± 0.14], broadly fusiform or oblong in side view, without suprahilar depression, adaxial side convex, apex blunt-round, oblong in front view; colorless, hyaline, smooth, slightly wall-thickened, dextrinoid, congophilous, not metachromatic in cresyl blue. ***Basidia*** 24–30 × 9–12 μm, clavate, mostly 4-spored and occasionally 2-spored, sterigmata up to 7 μm long. ***Cheilocystidia*** (Fig. 3C) 15–32 × 5–9 μm, diverse, clavate, fusiform, or falcate with flexuous apex, colorless, hyaline, wall-thinned, light pink in Congo red. ***Pleurocystidia*** absent. ***Pileus covering*** (Fig. 3D) a trichoderm composed of subcylindrical, narrow to apex, mostly flexuous, slightly wall-thickened, terminal elements 70–240 × 6–18 µm, base rarely with short elements, and with yellow to yellow-brownish intracellular pigment. ***Clamp connections*** present in all tissues.

##### Distribution.

Known only from the Xizang Autonomous Region and Sichuan Province, China.

##### Habitat.

Solitary or in small groups, saprotrophic and terrestrial on moist grasslands.

##### Additional specimens examined

**(paratypes).** China • Xizang: Jiangda County, Jila Mountain, 1 August 2008, alt. 4250 m, *Yang 4256* (HKAS45635); Sichuan: Daocheng County, alt. 4000 m, 1 July 1998, *Yang 1957* (HKAS32150).

##### Notes.

The main characteristics of *L. ochraceosquamea* are ochre-yellow squamules on the pileus, broadly fusiform basidiospores with a blunt-rounded apex, diverse shapes of cheilocystidia, and a trichodermal pileus covering with terminal elements gradually narrowing toward the apex, rarely with short elements at the base.

Phylogenetic analysis showed that *L. ochraceosquamea* clustered with several sequestrate and agaricoid species. Distinct from *L. ochraceosquamea*, the former species displays distinctly different characters, viz., sequestrate fruiting habit and globose basidiospores. The latter, comprising *L. albofloccosa*, *L. nigrosquamosa*, and *L. kuehneriana*, have distinct morphological features that differentiate them from *L. ochraceosquamea*. *Lepiota albofloccosa* has larger basidiomata, snow-white to milky white squamules on the pileus, longer [(11.5–)14.7–18.5(–21) × (5.5–)6.1–7.2(–8) μm] fusiform basidiospores, and a pileus covering with short elements at the base (Ahamed et al. 2023). *L. nigrosquamosa* differs in its medium-sized basidiomata, black-brownish to black squamules on the pileus, longer [(12)13–16 × 5–7 μm] basidiospores, and a pileus covering with basal short elements (Liang and Yang 2012). *Lepiota kuehneriana* possesses whitish, flocculent veil remnants on the margin of the pileus, longer basidiospores, and a pileus covering with terminal elements gradually narrowing toward the apex and short elements at the base (Liang 2007; Yang et al. 2019).

In addition, *L. aspericeps* Murrill also has yellowish-brown squamules on the pileus without striations; however, its longer (10.5–15.5 × 4.0–6.0 μm) basidiospores and a pileus covering with short elements at the base distinguish it from *L. ochraceosquamea* (Murrill 1951; Liang 2007).

## ﻿Discussion

Lepiota sect. Lepiota contains the type species *Lepiota clypeolaria* (Bull.) P. Kumm. and is the most diverse section in the genus. Vellinga (2001) characterized the section by spores that are fusiform-amygdaliform with convex abaxial and convex adaxial sides, or with a straight abaxial side, in combination with a pileus covering made up of long cylindrical elements often intermixed with short elements. In recent years, sequestrate *Lepiota* species have only been found in L. sect. Lepiota (Ge and Smith 2013; Lebel and Vellinga 2013; Vidal et al. 2015; Paz and Lavoise 2024). Such species with available sequences and formal descriptions were all sampled and phylogenetically located in Clades 1 and 2 (see Fig. 1). Except for *L. viridigleba* (Clade 2), which has fusiform basidiospores (Castellano 1995; Ge and Smith 2013), other sequestrate species (Clade 1) all possess globose to subglobose basidiospores. Sarawi et al. (2025) transferred *L. efibulis* Knudsen, *L. babruka* T.K.A. & Manim, and species of L. sect. Ovisporae subsect. FelinaeBon (1981) to L. sect. Lepiota. *L. efibulis* and *L. babruka* both have pyramidal scales on the pileus made up of globose to ellipsoid (oblong) elements in agglutinated chains and lacking clamp connections, which were originally placed in L. sect. Echinatae/*Echinoderma* s.l. (Knudsen 1980; Bon 1991; Vellinga 2001). The species of L. sect. Ovisporae subsect. Felinae have ellipsoid to oblong spores (Bon 1981; Candusso and Lanzoni 1990; Vellinga 2001; Sysouphanthong et al. 2011; Liang et al. 2018). In their study, *L. efibulis* and *L. babruka* were located in the basal subclade without Bayesian support, and species of L. sect. Ovisporae subsect. Felinae formed two separate subclades with high support values, which is consistent with our phylogeny. This treatment makes it difficult to determine the taxonomic position of the species of L. sect. Lepiota based on their morphological features, even when considering the diagnostic characters of the section. Further studies are necessary to better define diagnostic characters of the sections and the systematic position of *Lepiota* species.

*Lepiota pallidiochracea*, a species described from southwestern China with a trichodermial pileus covering and ellipsoid to oblong basidiospores measuring 9–12(14) × 6–8(9) μm, was placed in L. sect. Ovisporae but without phylogenetic analysis (Liang and Yang 2011; Yang et al. 2019). This species has larger basidiospores than those of L. sect. Ovisporae species (usually smaller than 10 μm), and our phylogenetic results indicated that it was nested within L. sect. Lepiota. Thus, *L. pallidiochracea* should be regarded as a member of L. sect. Lepiota.

Interestingly, our phylogenetic tree displays equal branch length of *L. eurysperma* and *L. pongduadensis* (Fig. 1), conflicting with the result of the original article (Sysouphanthong et al. 2012). A sequence alignment between the holotype (HQ718462) of *L. eurysperma* and the paratype (HQ718461) of *L. pongduadensis* showed that the two sequences had completely identical lengths and base arrangements. Morphological studies indicated that they were two different species (Sysouphanthong et al. 2012), while the fully consistent sequences suggest that one of the sequences is erroneous.

## Supplementary Material

XML Treatment for Lepiota brunneophora

XML Treatment for Lepiota ochraceosquamea

## ﻿Additional information

### Use of AI

No use of AI was reported.

### Author ORCIDs

Xing Li https://orcid.org/0000-0001-7146-0125

Bin Chen https://orcid.org/0000-0001-8459-3752

Yanliu Chen https://orcid.org/0000-0002-8054-2517

Mengya An https://orcid.org/0009-0001-6170-5886

Junfeng Liang https://orcid.org/0000-0002-0433-2577

### Data availability

All of the data that support the findings of this study are available in the main text or Supplementary Information.

## Supplementary materials

Supplementary material 1 Table of GenBank accession numbers Data type docx https://binary.pensoft.net/file/1437188This dataset is made available under the Open Database License (http://opendatacommons.org/licenses/odbl/1.0/). The Open Database License (ODbL) is a license agreement intended to allow users to freely share, modify, and use this Dataset while maintaining this same freedom for others, provided that the original source and author(s) are credited.Xing Li

Supplementary material 2 Alignment for phylogeny Data type fas https://binary.pensoft.net/file/1437189This dataset is made available under the Open Database License (http://opendatacommons.org/licenses/odbl/1.0/). The Open Database License (ODbL) is a license agreement intended to allow users to freely share, modify, and use this Dataset while maintaining this same freedom for others, provided that the original source and author(s) are credited.Xing Li
