# Supplementary material for: ﻿Multilocus phylogeny and morphology reveal two new species of Lepiota (Agaricales, Verrucosporaceae) from southwestern China
Source: MycoKeys. 2025 Oct 14;123:189–204. doi: 10.3897/mycokeys.123.163999 (PMC12541468; doi:10.3897/mycokeys.123.163999)
Supplement: Supplementary material 1 — Table of GenBank accession numbers [file mycokeys-123-189-s001.docx]

**Table 1.** A list of the vouchers, location and GenBank accession numbers for the sampled species in this study. The sequences obtained by new sequencing are indicated in bold, holotype and paratype are represented by HT and PT, respectively.

| **Taxon** | **Voucher** | **Location** | **Accession number** | | | | **References** |
| --- | --- | --- | --- | --- | --- | --- | --- |
|  |  |  | **ITS** | **LSU** | **IGS** | **mtSSU** |  |
| *L. alba* | HKAS90371 | China, Xinjiang | MN810115 | MN810075 |  |  | Hou and Ge 2020 |
| *L. albofloccosa* | MAA-01 (HT) | India | OP954870 | OP954873 |  |  | Ahamed et al. 2023 |
| *L. amplicystidiata* | HMAS53632 (PT) | China, Tibet | JN203141 |  |  | MK690180 | Liang 2012 |
| *L.* *angusticystidiata* | HKAS 50064 (HT) | China, Yunnan | KP177192 | KP177198 | MK705778 | KP177202 | Liang et al. 2018 |
| *L. aspericeps* | HKAS46051 | USA, Arkansas | **MK651620** |  | **MK705782** | **MK651711** | This study |
| *L. attenuate* | HKAS50110 (HT) | China, Yunnan | EU681776 | GU199354 | EU681801 | EU681826 | Liang et al. 2011 |
| *L. babruka* | K(M)155991 (HT) | India | PQ152691 |  |  | PQ152735 | Sarawi et al. 2025 |
| *L. brunneolilacea* | HKAS45634 | China, Tibet | **MK651607** | **MK651663** | **MK705772** | **MK651699** | This study |
| *L. brunneolilacea* | RITF617 | China, Yunnan | **MK651609** | **MK651664** | **MK705773** | **MK651700** | This study |
| *L. brunneophora* | RITF541 (PT) | China, Yunnan | **MK651649** | **MK685364** |  | **MK651736** | This study |
| *L. brunneophora* | RITF548 (HT) | China, Yunnan | **MK651650** | **MK685365** | **PV753061** | **MK651737** | This study |
| *L. clypeolaria* | HKAS46074 | China, Tibet | **MK651621** | **MK651670** | **EU681792** | **EU681814** | This study |
| *L. clypeolaria* | HKAS52854 | China, Yunnan | **MK651622** | **MK685363** | **MK705783** | **MK651713** | This study |
| *L. clypeolarioides* | FY010902L-oides48 | Japan, Yamanashi | LC688304 |  |  |  | Genbank, unpublished |
| *L. cortinarius* | HKAS46095 | China, Tibet | EU416306 | EU416307 | EU681798 | EU681823 | Liang et al. 2010 |
| *L. cremea* | OKA-TR11019 (HT) | Turkey, Denizli | OL630458 |  |  |  | Kaygusuz 2022 |
| *L. echinella* | 4-X-1998, H.A. Huijser | Belgium, Luxembourg | AY176366 | AY176367 |  |  | Vellinga 2004a |
| *L. efibulis* | K(M)155992 | India | PQ152706 |  |  | PQ152734 | Sarawi et al. 2025 |
| *L. efibulis* | K(M)188857 (HT) | UK, England | PQ152714 |  |  |  | Sarawi et al. 2025 |
| *L. elseae* | AH40487 (HT) | Spain | NR_158471 |  |  |  | Caballero et al. 2015 |
| *L. erminea* | SeSa98 | Germany | PP594552 | PP594670 |  | PQ152782 | Sarawi et al. 2025 |
| *L. eurysperma* | MFU0900035 (HT) | Thailand, Chiang Mai | HQ718462 |  |  |  | Sysouphanthong et al. 2012 |
| *L. faiae-bravae* | BCN-IC11111501 (HT) | Portugal, Guarda | PP622390 |  |  |  | Paz and Lavoise 2024 |
| *L. feline* | HKAS5801 | China, Jilin | EU416286 | EU416287 | EU681790 | EU681819 | Liang et al. 2010 |
| *L. forquignonii* | E.C. Vellinga 2284 (L) | Netherlands, Limburg | AY176370 | AY176371 |  |  | Vellinga 2004a |
| *L. forquignonii* | HMAS15448 | China, Heilongjiang | **MK651606** |  |  | **MK651698** | This study |
| *L. geocarpa* | UTC00143916 | USA, Utah | HQ020412 | EU130550 |  |  | Kropp et al. 2012 |
| *L. geogenia* | MEL:2358503 (PT) | Australia, Queensland | JX179267 | JX179271 |  |  | Lebel and Vellinga 2013 |
| *L. geogenia* | MEL:2358502 (HT) | Australia, Queensland | NR_120304 | JX179272 |  |  | Lebel and Vellinga 2013 |
| *L. geophana* | UTC00253060 (HT) | USA | HQ020411 | HQ020421 |  |  | Kropp et al. 2012 |
| *L. iberica* | JMV800332 (HT) | Spain | KT315646 |  |  |  | Vidal et al. 2015 |
| *L. ignivolvata* | SeSa97 | Germany | PP594551 | PP594669 |  | PQ152781 | Sarawi et al. 2025 |
| *L. kuehneriana* | HKAS45723 | China, Tibet | GU199360 | GU199358 | EU681797 | EU681825 | Liang et al. 2011 |
| *L. laevigata* | PAM14110916 | France | KT315652 |  |  |  | Vidal et al. 2015 |
| *L. lepida* | MCVE:727 | Unknown | FJ998392 |  |  |  | NCBI, unpublished |
| *L. maculans* | JMB080509_18 | USA, Tennessee | HM222939 | HQ832458 |  |  | Birkebak et al. 2011 |
| *L. magnispora* | HKAS8247 | China, Jilin | EU416288 | EU416289 | EU681793 | EU681815 | Liang et al. 2010 |
| *L. mandarina* | HKAS50028 (HT) | China, Yunnan | KM214811 | KM214816 | KM214818 | KM214821 | Liang 2016 |
| *L. mengei* | UTC00253524 (PT) | USA, Utah | MN810131 | MN810082 |  |  | Hou and Ge 2020 |
| *L. metulispora* | HMGID25584 | China | **MK651632** | **MK651674** | **MK705787** | **MK651721** | This study |
| *L. nigrosquamosa* | HKAS 33874 (HT) | China, Sichuan | JN203140 |  |  |  | Liang and Yang 2012 |
| *L. ochraceodisca* | PAM02111802 | France | KT315648 |  |  |  | Vidal et al. 2015 |
| *L. ochraceosquamea* | HKAS45635 (PT) | China, Tibet | **MK651633** | **MK651675** | **MK705788** | **MK651723** | This study |
| *L. ochraceosquamea* | HKAS45559 (HT) | China, Tibet | **MK651634** | **MK651676** |  | **MK651724** | This study |
| *L. oreadiformis* | SeSa96 | Germany | PP594550 | PP594668 |  | PQ152780 | Sarawi et al. 2025 |
| *L. oreadiformis* | SeSa21 | Austria | OL527686 | PP594637 |  |  | Sarawi et al. 2022, 2025 |
| *L. pakistanensis* | LAH37846 (HT) | Pakistan | OQ954776 | OQ954783 |  |  | Rehman et al. 2024 |
| *L. pallidiochracea* | HKAS45579 (HT) | China, Tibet | NR_158462 |  |  | **MK651718** | Liang and Yang 2011 |
| *L. pongduadensis* | MFU090184 (PT) | Thailand, Chiang Mai | HQ718461 |  |  |  | Sysouphanthong et al. 2012 |
| *L. pseudolilacea* | HKAS 8288 | China, Jilin | EU416304 | EU416305 | EU681804 | EU681808 | Liang et al. 2010 |
| *L. pseudolilacea* | E.C. Vellinga 2278 (L) | Netherlands, NoordHolland | AY176392 |  |  |  | Vellinga 2004a |
| *L. rufobrunnea* | HKAS52674 | USA, Arkansas | **MK651626** | **MK685362** | **MK705784** | **MK651715** | This study |
| *L. rufobrunnea* | MAA23-06 (HT) | India | PP331855 |  |  |  | Ahamed et al. 2024 |
| *L. sindhudeltana* | LAH37025 (HT) | Pakistan | OM987446 | OM987442 |  |  | Haqnawaz et al. 2022 |
| *L. smurfiorum* | JMV-8000331 (HT) | Spain | KT315647 |  |  |  | Vidal et al. 2015 |
| *L. spheniscispora* | E.C. Vellinga 2256 (HT) | USA, California | NR_119448 | AY176404 |  |  | Vellinga 2004a |
| *L. squamulodiffracta* | CA21 (HT) | Dominican, Sosua | NR_184876 |  |  |  | Justo et al. 2015 |
| *L. subcastanea* | HKAS45633 (HT) | China, Tibet | KM214812 | KM214817 | KM214819 | KM214820 | Liang 2016 |
| *L. subgracilis* | HKAS 5802 | China, Jilin | EU416290 | EU416291 | EU681795 | EU681811 | Liang et al. 2010 |
| *L. sublaevigata* | PAM02081205 | France | KT315650 |  |  |  | Vidal et al. 2015 |
| *L. thailandica* | MFLU 090120 (HT) | Thailand, Chiang Mai | JN224824 |  |  |  | Sysouphanthong et al. 2016 |
| *L. thailandica* | MFLU100516 (PT) | Thailand, Chiang Rai | KP348285 |  |  |  | Sysouphanthong et al. 2016 |
| *L. thrombophora* | HKAS41003 | China, Hainan | EU681780 | MK651687 | EU681800 | EU681813 | Liang et al. 2011 |
| *L. thrombophora* | RITF537 | China, Hainan | **MK651651** | **MK685366** | **MK705797** | **MK651738** | This study |
| *L. viridigleba* | OSC:56971 (HT) | Laos | NR_121542 |  |  |  | Ge and Smith 2013 |
| *L. xanthophylla* | E.C. Vellinga 2240 (L) | Netherlands, Limburg | AY176405 | AY176406 |  |  | Vellinga 2004a |

**Additional reference** (not included in the reference of text)

Ahamed M, Verma K, Dutta AK, Sharma YP (2024) A novel species of *Lepiota* sect. *Lepiota* (Agaricaceae) from Jammu and Kashmir, India. Taiwania 69(4): 522–529. https://doi.org/10.6165/tai.2024.69.522

Birkebak JM, Vellinga EC, Franco-Molano AE, Wood MG, Matheny PB (2011) *Lepiota* *maculans*, an Unusual Mushroom Rediscovered after 105 years. Southeastern Naturalist (Steuben, ME) 10(2): 267–274. https://doi.org/10.1656/058.010.0207

Caballero A, Vizzini A, Munoz G, Contu M, Ercole E (2015) *Lepiota* *elseae* (Agaricales, Agaricaceae), a new species of section *Lepiota* from Spain. Phytotaxa 201(3): 188–196. https://doi.org/10.11646/phytotaxa.201.3.2

Horak E (1980) On Australasian species of *Lepiota* S.F. Gray (Agaricales) with spurred spores. Sydowia 33: 111–144.

Hosen MI, Li TH, Ge ZW, Vellinga EC (2016) *Lepiota* *bengalensis*, a new species of *Lepiota* section *Lilaceae* from Bangladesh. Sydowia 68: 187–192. https://doi.org/10.12905/0380.sydowia68-2016-0187

Justo A, Angelini C, Bizzi A (2015) Two new species and a new record of *Lepiota* (Basidiomycota, Agaricales) from the Dominican Republic. Mycological Progress 14: 56(article number), 1–9. https://doi.org/10.1007/s11557-015-1080-9

Kropp BR, Albee-Scott S, Castellano MA, Trappe JM (2012) *Cryptolepiota*, a new sequestrate genus in the Agaricaceae with evidence for adaptive radiation in western North America. Mycologia 104(1): 164–174. https://doi.org/10.3852/11-046

Liang JF, Yang ZL, Xu JP, Ge ZW (2010) Two new unusual *Leucoagaricus* species (Agaricaceae) from tropical China with blue-green staining reactions. Mycologia 102(5): 1141–1152. https://doi.org/10.3852/09-021

Nawaz R, Khalid AN, Hanif M, Razaq A (2013) *Lepiota* *vellingana* sp. nov. (Basidiomycota, Agaricales) a new species from Lahore, Pakistan. Mycological Progress 12: 727–732. https://doi.org/10.1007/s11557-012-0884-0

Qasim T, Khalid AN, Vellinga EC, Razaq A (2015) *Lepiota* *albogranulosa* sp. nov. (Agaricales, Agaricaceae) from Lahore, Pakistan. Mycological Progress 14: 24(article number), 1–6. https://doi.org/10.1007/s11557-015-1037-z

Qasim T, Khalid AN, Vellinga EC (2016) A new species of *Lepiota*, *Lepiota* *lahorensis*, from Lahore, Pakistan. Turkish Journal of Botany 40(4): 419–426. https://doi.org/10.3906/bot-1507-31

Razaq A, Khalid AN, Ilyas S (2013) Molecular identification of *Lepiota* *acutesquamosa* and L. cristata (Basidiomycota, Agaricales) based on ITS-rDNA barcoding from Himalayan moist temperate forests of Pakistan. International Journal of Agriculture and Biology 15(2): 313–318.

Sysouphanthong P, Guo JY, Hyde KD, Xu JC, Mortimer PE (2016) *Lepiota* *thailandica* (Agaricaceae), a new species from Thailand. Phytotaxa 245(4): 262–270. https://doi.org/10.11646/phytotaxa.245.4.3
